# Clipping the Positive Lymph Node in Patients with Clinically Node Positive Breast Cancer Treated with Neoadjuvant Chemotherapy: Impact on Axillary Surgery in the ISPY-2 Clinical Trial

**DOI:** 10.1245/s10434-024-15792-x

**Published:** 2024-07-12

**Authors:** Kayla M. Switalla, Judy C. Boughey, Katrina Dimitroff, Christina Yau, Velle Ladores, Hongmei Yu, Julia Tchou, Mehra Golshan, Gretchen Ahrendt, Lauren M. Postlewait, Mara Piltin, Chantal R. Reyna, Cindy B. Matsen, Todd M. Tuttle, Anne M. Wallace, Cletus A. Arciero, Marie Catherine Lee, Jennifer Tseng, Jennifer Son, Roshni Rao, Candice Sauder, Arpana Naik, Marissa Howard-McNatt, Rachael Lancaster, Peter Norwood, Laura J. Esserman, Rita A. Mukhtar

**Affiliations:** 1 University of Minnesota Medical School, Minneapolis, MN USA; 2 Department of Surgery, University of California San Francisco, San Francisco, CA USA; 3 Department of Surgery, Mayo Clinic, Rochester, MN USA; 4 Quantum Leap Healthcare Collaborative, San Francisco, CA USA; 5 Department of Surgery, University of Pennsylvania, Philadelphia, PA USA; 6 Department of Surgery, Yale School of Medicine, New Haven, CT USA; 7 Department of Surgery, University of Colorado, Aurora, CO USA; 8 Department of Surgery, Emory University, Atlanta, GA USA; 9 Department of Surgery, Loyola University Medical Center, Chicago, IL USA; 10 Department of Surgery, University of Utah, Salt Lake City, UT USA; 11 Department of Surgery, University of Minnesota, Minneapolis, MN USA; 12 Department of Surgery, University of California San Diego, San Diego, CA USA; 13 Department of Surgery, Moffitt Cancer Center, Tampa, FL USA; 14 Department of Surgery, City of Hope, Irvine, CA USA; 15 Department of Surgery, MedStar Georgetown University, Washington, DC USA; 16 Department of Surgery, Columbia University Medical Center, New York, NY USA; 17 Department of Surgery, UC Davis Health Comprehensive Cancer Center, Sacramento, CA USA; 18 Department of Surgery, Oregon Health and Science University, Portland, OR USA; 19 Department of Surgery, Wake Forest School of Medicine, Winston-Salem, NC USA; 20 Division of Surgical Oncology, The University of Alabama at Birmingham Medical Center, Birmingham, AL USA; 21 UCSF Breast Care Center, San Francisco, CA USA

**Keywords:** Neoadjuvant chemotherapy, Breast cancer, Clipped node, Sentinel lymph node surgery, Targeted axillary dissection

## Abstract

**Background:**

For patients with clinically node-positive (cN+) breast cancer undergoing neoadjuvant chemotherapy (NAC), retrieving previously clipped, biopsy-proven positive lymph nodes during sentinel lymph node biopsy [i.e., targeted axillary dissection (TAD)] may reduce false negative rates. However, the overall utilization and impact of clipping positive nodes remains uncertain.

**Patients and Methods:**

We retrospectively analyzed cN+ ISPY-2 patients (2011–2022) undergoing axillary surgery after NAC. We evaluated trends in node clipping and associations with type of axillary surgery [sentinel lymph node (SLN) only, SLN and axillary lymph node dissection (ALND), or ALND only] and event-free survival (EFS) in patients that were cN+ on a NAC trial.

**Results:**

Among 801 cN+ patients, 161 (20.1%) had pre-NAC clip placement in the positive node. The proportion of patients that were cN+ undergoing clip placement increased from 2.4 to 36.2% between 2011 and 2021. Multivariable logistic regression showed nodal clipping was independently associated with higher odds of SLN-only surgery [odds ratio (OR) 4.3, 95% confidence interval (CI) 2.8–6.8, *p* < 0.001]. This was also true among patients with residual pathologically node-positive (pN+) disease. Completion ALND rate did not differ based on clip retrieval success. No significant differences in EFS were observed in those with or without clip placement, both with or without successful clip retrieval [hazard ratio (HR) 0.85, 95% CI 0.4–1.7, *p* = 0.7; HR 1.8, 95% CI 0.5–6.0, *p* = 0.3, respectively].

**Conclusion:**

Clip placement in the positive lymph node before NAC is increasingly common. The significant association between clip placement and omission of axillary dissection, even among patients with pN+ disease, suggests a paradigm shift toward TAD as a definitive surgical management strategy in patients with pN+ disease after NAC.

Breast cancer management typically requires an assessment of axillary lymph node status to inform prognosis and guide therapeutic decision making. Historically, axillary lymph node dissection (ALND) was the standard of care for axillary staging but is associated with significant morbidity, including a 6–30% risk of lymphedema.^1–3^ Over the last two decades, several prospective, randomized clinical trials have demonstrated the reliability of using sentinel lymph node (SLN) surgery as a less morbid means for nodal staging in patients with clinically node negative (cN0) disease without impacting recurrence or survival when compared with ALND.^4–7^ There has consequently been a notable reduction in the use of ALND, with SLN surgery being utilized for axillary staging in patients with cN0 disease in both in the setting of upfront surgery and after neoadjuvant chemotherapy (NAC).^4,7–11^

However, for patients with cN+ disease who proceed to upfront surgery and for those who have residual nodal disease after NAC, current guidelines recommend ALND.^8^ Since advances in systemic therapy have led to higher rates of complete pathologic nodal response after NAC, there is increasing interest in accurately identifying patients with cN+ disease who have eradication of nodal disease (i.e., convert to pN0) and can be spared ALND. While several studies have shown that traditional SLN surgery in patients with cN+ disease after NAC results in false negative rates (FNR) exceeding 10%, several techniques have been identified that may mitigate this problem.^12–14^ Specifically, the ACOSOG Z1071 trial showed that a FNR < 10% can be achieved with the use of dual tracer and excision of ≥ 3 SLNs.^12^

An additional technique for reducing the FNR of SLN surgery after NAC in patients with cN+ disease includes placement of a clip in the biopsy-proven positive lymph node prior to NAC and subsequent resection at the time of SLN surgery.^15^ This combination of SLN surgery and removal of the previously clipped node has been termed targeted axillary dissection (TAD) and has been shown to be associated with FNRs as low as 2.4%.^16^ Findings from the recent, prospective SenTa study suggest that TAD without ALND confers similar survival and recurrence outcomes compared with TAD with ALND^17^, and further data from prospective trials are awaited regarding the optimal management of pathologic node positive disease after NAC. The implementation of lymph node clipping and subsequent localization and resection of the clipped node is inconsistent, however, and its utility has been debated.^18,19^

In this study, we analyzed data from the ISPY-2 trial, a prospective, randomized, multicenter NAC trial to understand the prevalence of nodal clipping and associated axillary surgical procedures. While the ISPY-2 trial protocol includes recommendations for axillary management, the specific surgical approach is not mandated, making these data a unique representation of nodal clipping practices across 24 medical centers in the USA, in the context of a trial that includes careful preoperative imaging (with serial breast magnetic resonance imaging) and thorough pathologic assessment of surgical specimens.

## Patients and Methods

### Study Population and Data Collection

ISPY-2 is a multicenter, randomized neoadjuvant chemotherapy trial for patients with molecularly high-risk breast cancer (NCT01042379). Per protocol, patients are randomized to neoadjuvant novel systemic therapy agents followed by surgical resection, with pathologic complete response (pCR) rates being the primary study endpoint. While type of breast and axillary surgery is not dictated by the trial, recommended standards for axillary management within the trial have been published and include the following: axillary ultrasound and percutaneous needle biopsy of the most abnormal node, if abnormal nodes are present, is required. For patients with cN+ disease, SLN surgery is permitted but requires use of dual tracer, with resection of all sentinel nodes and removal of at least two nodes required if no clip was placed. Placement of a clip in the positive axillary node in patients with node positive disease and preoperative localization of the clipped node is strongly recommended but not required; for those patients with a clipped node, resection of the clipped node should be performed. In instances of pathological positive node(s), additional axillary surgery is not obligated and is left to the discretion of the treating surgeon in both cN0 and cN+ groups.^10,20^ Of note, in rare instances where axillary management guidelines are not met, patients are still evaluated on trial; however, in the unusual occurrence of patients undergoing no axillary surgery at all, the primary efficacy endpoint is administratively considered a non-pCR, since pCR status cannot be ascertained.

We retrospectively analyzed all patients with cN+ breast cancer who were enrolled in ISPY-2 from 01/2011 to 12/2021. Clinical node positivity was determined by percutaneous needle biopsy (fine needle aspiration or core needle biopsy) of an abnormal axillary node prior to NAC. All included patients received NAC followed by axillary surgery, categorized as either SLN-only surgery, SLN and axillary dissection (ALND), or ALND-only, based on procedures defined in operative reports. We collected baseline clinicopathologic features, including age, self-reported race/ethnicity, tumor receptor subtype, clinical T (cT) category at diagnosis, clinical N (cN) category at diagnosis, pathologic T (pT) category, pathologic N (pN) category, residual cancer burden (RCB) class, year and type of axillary surgery, nodal clip placement, and number of nodes removed. Patients were categorized into one of two groups: those with clip placement in the biopsy-proven axillary lymph node prior to NAC and those without clip placement in an axillary lymph node prior to NAC. Clip localization method was determined by review of operative, pathology, and/or imaging reports. Successful clip retrieval was determined by confirmation of the clip and/or clip localization device being removed at the time of surgery within any operative reports, pathology reports, and/or postoperative hospital visit records. For cases in which there was no mention of clip or localization device removal at the time of surgery, these cases were categorized as “No mention of clip removal” and were excluded from analyses of clip retrieval rates. For cases in which there was confirmation of a localization device being removed but no mention of whether or not the clip was retrieved (*n* = 18), we assumed these cases to have successful clip retrieval. Event-free survival (EFS) was defined as patient survival without local or distant breast cancer recurrence or death; patients without a recurrence event or death were censored at the date of last follow-up.

We specifically investigated (1) what proportion of cN+ patients had a clip placed in an axillary lymph node prior to NAC and whether this has changed over time, (2) whether patients with a clip placed in a lymph node underwent different axillary surgery procedures compared with those without a clip placed, and (3) whether clipping a node is associated with higher rates of completion axillary dissection, particularly in those for whom the clip is not retrieved. Finally, we investigated EFS in those with and without nodal clip placement, as well as in those with and without successful clip retrieval.

### Statistical Analyses

Demographic information and clinicopathologic features were compared between patients with and without a clipped node using Pearson’s chi-squared test, Fisher’s exact test, and Wilcoxon rank-sum test. A multivariable logistic regression model was developed to assess the influence of lymph node clipping on use of SLN only, adjusted for surgery year and cN category. Event-free survival was evaluated using the log-rank test and Kaplan–Meier survival analysis. Univariable and multivariable Cox proportional hazards models were used to assess hazard ratios (HRs) with 95% confidence intervals (CIs). Statistical analyses were performed using R version 4.3.1. Two-tailed *p* values < 0.05 were considered statistically significant.

## Results

### Clinicodemographic Information and Trends in Lymph Node Clipping

**Fig. 1:**
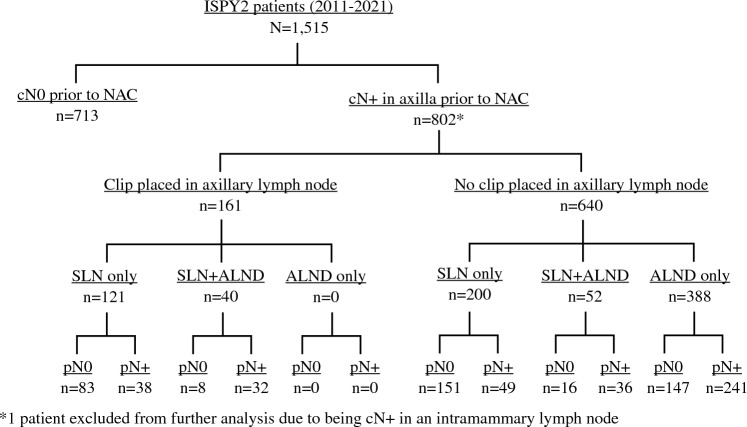
Patient distribution by cN status, use of clip placement in an axillary node, and type of axillary surgery. cN0 clinically node negative, cN+ clinically node positive, NAC neoadjuvant chemotherapy, SLN sentinel lymph node, ALND axillary lymph node dissection, pN0 pathologically node negative, pN+ pathologically node positive

Between 2011 and 2021, there were 1515 patients enrolled in ISPY-2 who completed NAC and surgery, with 802 (52.9%) identified as cN+. One cN+ case was excluded from our analyses due to being cN+ in an intramammary node but missing axillary nodal status. After this exclusion, our final cohort consisted of 801 patients with clinical node positivity in the axilla (Fig. 1). Average age ± standard deviation (SD) of the 801 patients with cN+ disease was 48.1 ± 11.3 years. Tumor receptor subtype was hormone receptor (HR) positive and HER2 negative in 50.0% of cases (401 patients), HER2 positive in 22.6% (181 patients), and HR negative and HER2 negative in 27.3% (219 patients). The majority of patients with cN+ disease had cN1 disease (80.8%), 8.2% had cN2 disease, and 11% had cN3 disease at presentation.

Among these 801 cN+ patients, 161 (20.1%) had a clip placed in a biopsy-proven positive axillary node prior to NAC, while 640 (79.9%) did not. The proportion of patients with cN+ disease undergoing clip placement increased over time from 2.4% in 2011 to 36.2% in 2021 (Table 1). Compared with those without clipped nodes, the clipped node cohort had a higher proportion of patients with cT1 (3.1% versus 0.5%) and cT2 disease (66% versus 58%), and a higher proportion with residual cancer burden (RCB) class 0 (42% versus 30%) (Table 1). There was no difference in age, tumor receptor subtype, or type of breast surgery performed between the clipped and nonclipped cohorts.

**Table 1:** Characteristics and clinicopathologic features of cN+ patients with and without clipped nodes

		Use of nodal clipping		
Characteristic	Overall, *N* = 801^a^	Clipped node, *N* = 161^a^	No clipped node, *N* = 640^a^	*p* value^b^
Age	48.09 (11.28)	46.94 (11.45)	48.38 (11.22)	0.2
Surgery year				< 0.001
2011	41/801 (5.1%)	1/41 (2.4%)	40/41 (98%)	
2012	59/801 (7.4%)	1/59 (1.7%)	58/59 (98%)	
2013	70/801 (8.7%)	6/70 (8.6%)	64/70 (91%)	
2014	74/801 (9.2%)	6/74 (8.1%)	68/74 (92%)	
2015	78/801 (9.7%)	10/78 (13%)	68/78 (87%)	
2016	62/801 (7.7%)	11/62 (18%)	51/62 (82%)	
2017	78/801 (9.7%)	15/78 (19%)	63/78 (81%)	
2018	76/801 (9.5%)	16/76 (21%)	60/76 (79%)	
2019	114/801 (14%)	35/114 (31%)	79/114 (69%)	
2020	80/801 (10.0%)	35/80 (44%)	45/80 (56%)	
2021	69/801 (8.6%)	25/69 (36%)	44/69 (64%)	
Race				0.069
Asian	53/801 (6.6%)	14/161 (8.7%)	39/640 (6.1%)	
Black	103/801 (13%)	12/161 (7.5%)	91/640 (14%)	
Other	15/801 (1.9%)	2/161 (1.2%)	13/640 (2.0%)	
White	630/801 (79%)	133/161 (83%)	497/640 (78%)	
Ethnicity				0.6
Hispanic or Latino	98/801 (12%)	17/161 (11%)	81/640 (13%)	
Not Hispanic or Latino	699/801 (87%)	144/161 (89%)	555/640 (87%)	
Unknown	4/801 (0.5%)	0/161 (0%)	4/640 (0.6%)	
Tumor subtype				0.056
HR+HER2−	401/801 (50%)	88/161 (55%)	313/640 (49%)	
HR−HER2−	219/801 (27%)	48/161 (30%)	171/640 (27%)	
HER2+	181/801 (23%)	25/161 (16%)	156/640 (24%)	
Clinical T category at diagnosis				0.001
T1	8/801 (1.0%)	5/161 (3.1%)	3/640 (0.5%)	
T2	477/801 (60%)	106/161 (66%)	371/640 (58%)	
T3	272/801 (34%)	47/161 (29%)	225/640 (35%)	
T4	44/801 (5.5%)	3/161 (1.9%)	41/640 (6.4%)	
Clinical N category at diagnosis				0.082
N0	0/801 (0%)	0/161 (0%)	0/640 (0%)	
N1	647/801 (81%)	140/161 (87%)	507/640 (79%)	
N2	66/801 (8.2%)	8/161 (5.0%)	58/640 (9.1%)	
N3	88/801 (11%)	13/161 (8.1%)	75/640 (12%)	
Pathologic T category				0.036
T0	236 /798 (30%)	62/160 (39%)	174/638 (27%)	
T1	240/798 (30%)	45/160 (28%)	195/638 (31%)	
T2	153/798 (19%)	29/160 (18%)	124/638 (19%)	
T3	93/798 (12%)	13/160 (8.1%)	80/638 (13%)	
T4	14/798 (1.8%)	0/160 (0%)	14/638 (2.2%)	
Tis	62/798 (7.8%)	11/160 (6.9%)	51/638 (8.0%)	
Unknown	3	1	2	
Pathologic N category				0.008
N0	405/801 (51%)	91/161 (57%)	314/640 (49%)	
N1	253/801 (32%)	56/161 (35%)	197/640 (31%)	
N2	99/801 (12%)	11/161 (6.8%)	88/640 (14%)	
N3	44/801 (5.5%)	3/161 (1.9%)	41/640 (6.4%)	
RCB class				<0.001
0	260/801 (32%)	68/161 (42%)	192/640 (30%)	
1	100/801 (12%)	17/161 (11%)	83/640 (13%)	
2	273/801 (34%)	60/161 (37%)	213/640 (33%)	
3	168/801 (21%)	16/161 (9.9%)	152/640 (24%)	
Type of breast surgery				0.12
Partial mastectomy	281/734 (38%)	54/139 (39%)	227/595 (38%)	
Nipple-sparing mastectomy	107/734 (15%)	25/139 (18%)	82/595 (14%)	
Skin-sparing mastectomy	111/734 (15%)	26/139 (19%)	85/595 (14%)	
Total mastectomy	235/734 (32%)	34/139 (24%)	201/595 (34%)	
Unknown	67	22	45	
Type of axillary surgery				<0.001
SLN Only	321/801 (40%)	121/161 (75%)	200/640 (31%)	
ALND and SLN	92/801 (11%)	40/161 (25%)	52/640 (8.1%)	
ALND only	388/801 (48%)	0/161 (0%)	388/640 (61%)	
Average total number of nodes removed at surgery	11.37 (8.81)	6.89 (6.57)	12.50 (8.95)	< 0.001
SLN only subset	*N* = 321 4.15 (2.91)	*N* = 121 4.21 (3.14)	*N* = 200 4.11 (2.77)	0.9
ALND and SLN subset	*N* = 92 15.52 (8.27)	*N* = 40 14.98 (7.59)	*N* = 52 15.94 (8.81)	0.8
ALND only subset	*N* = 388 16.37 (8.07)	*N* = 0 0 (0)	*N* = 388 16.37 (8.07)	–
Total positive nodes removed at surgery	2.12 (4.08)	1.27 (2.44)	2.34 (4.37)	0.005
Number of positive sentinel nodes removed	0.51 (1.69)	0.76 (1.09)	0.45 (1.80)	< 0.001

Data expressed as mean (SD) or* n*/*N* (%).

*HR* hormone receptor, *HER2* human epidermal growth factor receptor 2, *RCB* residual cancer burden, *SLN* sentinel lymph node, *ALND* axillary lymph node dissection

^a^Mean (SD); *n*/*N* (%)

^b^Wilcoxon rank-sum test; Pearson’s chi-squared test; Fisher’s exact test

### Associations between Clip Placement and Type of Axillary Surgery

Overall, axillary surgery performed was SLN only in 40.1%, SLN and ALND in 11.5%, and ALND only in 48.4% (Table 1). On univariate analysis, the clipped node cohort had a significantly higher proportion of those who underwent SLN-only surgery (75.2% versus 31.2%, *p* < 0.001) and a concomitant lower proportion undergoing ALND-only surgery (0% versus 60.6%, *p* < 0.001) compared with the nonclipped node cohort (Table 1). Clip placement remained associated with a significantly higher rate of SLN-only surgery compared with no clip placement within each cN category (75% versus 34% for cN1, *p* < 0.001; 63% versus 21% for cN2, *p* = 0.023; 85% versus 23% for cN3, *p* < 0.001) (Table 2). When adjusted for both year of surgery and cN category, multivariable logistic regression showed that nodal clipping was independently associated with higher odds of SLN-only surgery [odds ratio (OR) 4.3, 95% CI 2.8–6.8, *p* < 0.001].

**Table 2:** Type of axillary surgery by clip placement and by cN category

		Type of axillary surgery		
Variable	*N*	All other axillary surgery, *N* = 371^a^	SLN surgery only, *N* = 276^a^	*p* value^b^
Patients with cN1				
Nodal clip placement	647			< 0.001
No clip placed		336/507 (66%)	171/507 (34%)	
Clip placed		35/140 (25%)	105/140 (75%)	
Patients with cN2				
Nodal clip placement	66			0.023
No clip placed		46/58 (79%)	12/58 (21%)	
Clip placed		3/8 (38%)	5/8 (63%)	
Patients with cN3				
Nodal clip placement	88			< 0.001
No clip placed		58/75 (77%)	17/75 (23%)	
Clip placed		2/13 (15%)	11/13 (85%)	

^a^*n*/*N* (%)

^b^Pearson’s chi-squared test

Clip placement remained associated with SLN-only surgery even among patients who remained pathologically node positive (pN+). Among the 161 patients in the clipped node cohort, pN+ disease was found in 70 (43.5%). Of those 70 patients with pN+ disease, axillary surgical management was SLN only in 54.3%, SLN and ALND in 45.7%, and ALND only in none. Among the 640 patients in the nonclipped node cohort, pN+ disease was found in 326 (50.9%). Of those 326 patients with pN+ disease, surgical management of the axilla was SLN only in 15%, SLN and ALND in 11%, and ALND only in 74%. Patients with pN+ in the clipped node group were significantly more likely to undergo SLN-only surgery compared with patients with pN+ in the nonclipped node cohort (54.3% versus 15.0%, *p* < 0.001).

### Clip Localization and Retrieval Rates

Clip localization method and retrieval status were available in 147 of 161 clipped node cases (91.3%). The overall clip retrieval rate was 85.7% (126/147) and the preoperative clip localization rate was 73.5% (108/147). Use of clip localization was associated with a higher rate of successful retrieval of the clipped node compared with procedures done without localization (96% versus 56%, *p* < 0.001). When stratified by type of localization, successful retrieval of the clipped node did not differ by localization method (MagSeed: 25/25, 100%; radioactive seed: 29/30, 97%; Savi Scout 32/33, 97%; wire 18/20, 90%; *p* = 0.356). The rate of completion axillary dissection did not differ between cases with successful clip retrieval (*n* = 126) compared with those without successful clip retrieval (*n* = 21) (completion axillary dissection rate of 24.6% versus 23.8%, respectively, *p* > 0.9). Of the clipped node patients who underwent completion axillary dissection, 16.1% in the clip-retrieval group were pN0, and 40% of those without clip retrieval were pN0. In the overall subset of patients with clip placement who converted to pN0 status (*n* = 83), there was no difference in the rate of completion axillary dissection among those with clip retrieval versus no clip retrieval (7.2% versus 14.3%, *p* = 0.3).

### Number of Nodes Removed in SLN-Only Cohort

Among the 321 patients who underwent SLN-only surgery, the average number of nodes excised did not differ between clipped node (*n* = 121) and nonclipped node (*n* = 200) patients (mean 4.2 versus 4.1 nodes, *p* = 0.9). However, within the clipped node cohort, the use of clip localization (i.e., TAD) was associated with greater number of lymph nodes removed compared with those undergoing SLN-only surgery without clip localization (mean 4.6 versus 3.4 nodes, *p* = 0.011).

### Event-Free Survival in cN+ Cohort

With mean follow-up time of 3.5 years (standard deviation 1.9), there were 146 patients with events, including 49 local recurrences (of which 12 cases involved axillary recurrence), 109 distant recurrences, and 80 deaths. Of those 12 patients who experienced axillary recurrence, 33.3% had undergone SLN only, 6.7% had undergone SLN and ALND, and 50.0% had undergone ALND. By pathologic nodal response, the axillary recurrence rate was 0.7% in patients with ypN0 disease and 2.3% in patients with ypN+ disease (*p* = 0.074).

**Fig. 2:**
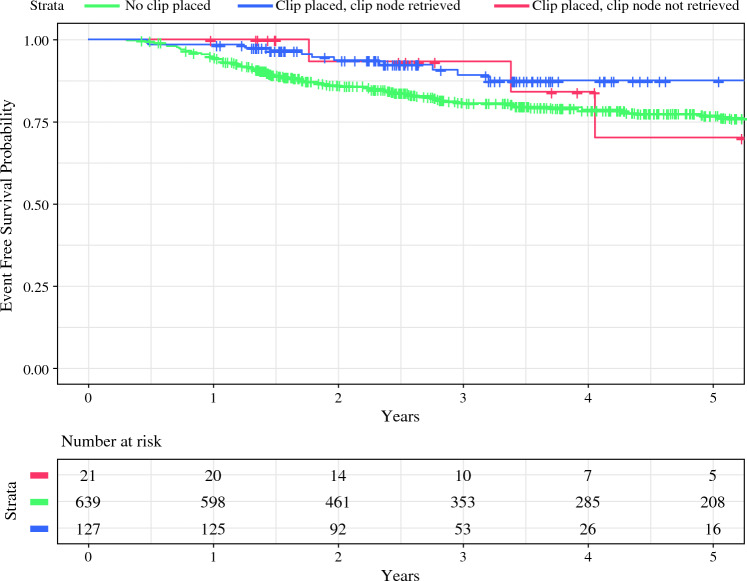
Kaplan-Meier survival analysis for event-free survival (EFS) based on clip placement and clip retrieval status

In unadjusted evaluation of EFS at 5 years by log-rank test, the cumulative estimated EFS was 77% for those without clip placement, 87% for those with a clip placed and successfully retrieved, and 70% for those with a clip placed but not retrieved (*p* = 0.053) (Fig. 2). In a multivariable Cox proportional hazards model adjusted for age, race/ethnicity, tumor receptor subtype, cT category, cN category, residual cancer burden (RCB) class, year of surgery, type of breast and axillary surgery, and number of nodes removed, there was no significant difference in EFS between those without clip placement compared with those with (HR 0.7, 95% CI 0.4–1.7, *p* = 0.7) or without (HR 1.8, 95% CI 0.5–6.0, *p* = 0.3) successful clip retrieval (Table 3).

**Table 3:** Univariable and multivariable Cox proportional hazards model for event-free survival (EFS) in cN+ patients with and without a clipped node

	EFS univariate analysis					EFS multivariate analysis		
Characteristic	*N*	Event *N*	HR^a^	95% CI^a^	*p* value^b^	HR^a^	95% CI^a^	*p* value^b^
Clip placement								
No clip placed	639	128	–	–		–	–	
Clip placed and clipped node retrieved	127	11	0.48	0.26, 0.89	**0.019**	0.85	0.42, 1.74	0.7
Clip placed, but clipped node not retrieved	21	3	0.76	0.24, 2.38	0.6	1.80	0.54, 6.01	0.3
Age at screening	801	146	1.00	0.98, 1.01	0.5	1.00	0.98, 1.01	0.8
Surgery year								
2011	41	13	–	–		–	–	
2012	59	12	0.65	0.30, 1.43	0.3	0.66	0.29, 1.50	0.3
2013	70	17	0.80	0.39, 1.65	0.5	1.05	0.50, 2.19	0.9
2014	74	16	0.71	0.34, 1.47	0.4	0.56	0.26, 1.19	0.13
2015	78	17	0.73	0.36, 1.51	0.4	0.69	0.33, 1.46	0.3
2016	62	12	0.68	0.31, 1.49	0.3	0.59	0.26, 1.37	0.2
2017	78	17	0.80	0.39, 1.66	0.6	0.78	0.36, 1.68	0.5
2018	76	13	0.72	0.33, 1.56	0.4	0.74	0.33, 1.66	0.5
2019	114	14	0.53	0.25, 1.13	0.10	0.72	0.32, 1.63	0.4
2020	80	7	0.46	0.18, 1.17	0.10	0.62	0.22, 1.71	0.4
2021	69	8	0.86	0.35, 2.13	0.8	0.00	0.00, inf.	> 0.9
Race								
White	630	113	–	–		–	–	
Asian	53	8	0.81	0.40, 1.67	0.6	0.62	0.29, 1.33	0.2
Black	103	24	1.35	0.87, 2.10	0.2	1.24	0.75, 2.04	0.4
Other	15	1	0.41	0.06, 2.94	0.4	1.27	0.17, 9.52	0.8
Ethnicity								
Not Hispanic or Latino	699	130	–	–		–	–	
Hispanic or Latino	98	16	0.92	0.55, 1.55	0.8	1.22	0.69, 2.16	0.5
Unknown	4	0	0.00	0.00, Inf	> 0.9	0.00	0.00, inf.	> 0.9
Clinical T Category at diagnosis								
T1/T2	485	67	–	–		–	–	
T3/T4	316	79	2.04	1.47, 2.82	**< 0.001**	1.52	1.05, 2.21	**0.026**
Clinical N category at diagnosis								
N1	647	102	–	–		–	–	
N2	66	25	2.78	1.80, 4.31	**<0.001**	2.77	1.72, 4.47	**< 0.001**
N3	88	19	1.49	0.91, 2.43	0.11	2.08	1.20, 3.60	**0.009**
Tumor receptor subtype								
HR+ HER2–	401	68	–	–		–	–	
HER2+	181	20	0.59	0.36, 0.97	**0.037**	1.11	0.64, 1.91	0.7
HR– HER2–	219	58	1.70	1.20, 2.42	**0.003**	2.72	1.83, 4.05	**< 0.001**
RCB class								
0	260	14	–	–		–	–	
1	100	15	2.89	1.40, 5.99	**0.004**	2.29	1.02, 5.15	**0.045**
2	273	60	4.52	2.53, 8.09	**<0.001**	5.89	3.11, 11.2	**< 0.001**
3	168	57	7.55	4.21, 13.6	**<0.001**	8.55	4.39, 16.6	**< 0.001**
Type of axillary surgery								
All other axillary surgery	480	112	–	–		–	–	
SLN surgery only	321	34	0.48	0.33, 0.71	**<0.001**	0.67	0.38, 1.18	0.2
Type of breast surgery								
Partial mastectomy	281	39	–	–		–	–	
Nipple sparing mastectomy	107	17	1.21	0.69, 2.15	0.5	1.14	0.62, 2.08	0.7
Skin sparing mastectomy	111	19	1.30	0.75, 2.25	0.3	0.98	0.53, 1.82	> 0.9
Total mastectomy	235	62	1.97	1.32, 2.94	**< 0.001**	1.35	0.85, 2.14	0.2
Total Nodes Removed	801	146	1.02	1.01, 1.04	**0.009**	0.99	0.96, 1.01	0.3

*HR* hazard ratio, *CI* confidence interval, *HR* hormone receptor, *HER2* human epidermal growth factor receptor 2, *RCB* residual cancer burden, *SLN* sentinel lymph node

^a^*HR* hazard ratio, *CI* confidence interval

^b^Wald test

## Discussion

In this analysis evaluating patients with cN+ disease treated with NAC on the ISPY-2 trial, we found that rates of nodal clipping have increased significantly over the last decade and this practice is associated with increased use of SLN surgery, independent of surgery year and clinical nodal burden. Importantly, the clipped node cohort had significantly lower rates of axillary dissection than the nonclipped node cohort, even in the setting of residual nodal disease. Additionally, patients with clipped nodes had a comparable number of nodes removed at SLN surgery/TAD compared with those without a clip placed, and there was no difference in EFS between clipped and nonclipped node patients. Overall, these findings reflect a paradigm shift in the management of the axilla, suggesting that surgeons are increasingly utilizing TAD as definitive surgical management of the axilla rather than as a staging procedure in the post-NAC setting.

TAD was initially conceived as a staging procedure to address limitations in the accuracy of SLN biopsy alone for patients with cN+ disease after NAC. Since 2013, several prospective trials have evaluated the accuracy of SLN surgery after NAC in patients with cN+. The initial key trials demonstrated FNRs ranging from 8.4 to 14.2%.^12–14^ In recent years, the use of clip placement prior to NAC to facilitate identification and resection of the biopsy proven lymph node at time of surgery has been proposed as a means of reducing the FNR of SLN surgery after NAC. Specifically, the term TAD has been coined to describe cases where the index positive node is clipped during biopsy and subsequently localized preoperatively in addition to SLN surgery. However, use of TAD has been inconsistent.^15,16,21^

Our study showed that both clip placement and SLN surgery have significantly increased over time in patients with cN+ disease and that clip placement was independently associated with SLN-only surgery overall and within each cN category. Additionally, we found that among patients with pN+ disease after NAC, axillary dissection was omitted in 54% of the clipped node group, compared with only 15% of the nonclipped node group. These findings may reflect the fact that surgeons who adopted the practice of node clipping also adopted SLN surgery after NAC; however, the high rates of omitting axillary dissection in patients with pN+ disease who underwent TAD suggest that clip placement may have been pursued with the intention of limiting axillary surgery regardless of nodal response. While not yet the standard of care, such approaches are increasingly employed in the effort to minimize the morbidity of axillary management and reduce the risk of lymphedema, particularly in the setting of data suggesting that tumor biology and response are more strongly related to recurrence risk than extent of surgery.^22^ This approach has been described as “tailored axillary surgery,” with the goal of selectively removing positive nodes to achieve residual disease burden that can be controlled with radiotherapy.^23^ The ongoing prospective, multicenter, international TAXIS trial is currently testing whether tailored axillary surgery with nodal radiation is noninferior to ALND with nodal radiation in patients with nodal disease in both the adjuvant and post-neoadjuvant settings.^23,24^

In our analysis we note that clips were more likely to be placed in patients who had lower cT category disease at diagnosis. This observation suggests that the decision to place clips may be influenced by pre-NAC staging factors. Additionally, patients with a greater response to NAC, evidenced by lower RCB, were also more likely to have clips placed at diagnosis. However, it is important to acknowledge that the decision to place clips is typically made prior to the onset of NAC. Therefore, while these associations may suggest a selective use of clip placement in patients who were anticipated to potentially avoid ALND after NAC, we cannot conclude causality.

Reassuringly, we found no difference in the number of nodes removed during SLN surgery for clipped versus nonclipped patients. While the ISPY-2 trial guidelines recommend localization of clipped nodes prior to surgery, the specific localization method is not mandated and not all patients with clipped nodes had preoperative localization performed. We found that the method of localization was not associated with differential rates of successful clip retrieval. When clip localization was used, the clipped node was more likely to be retrieved, at the cost of a slightly but significantly higher number of total nodes removed (mean of 4.6 versus 3.4 nodes, *p* = 0.011). One hypothesis for this difference in number of lymph nodes removed is that when localization is used, surgeons are more likely to continue to search for additional nodes to ensure specific removal of the localizer and the clipped node. Alternatively, to avoid missing the localized node, surgeons may be resecting a larger specimen that includes the marked lymph node and additional adjacent nodes leading to a higher number of nodes resected in patients with clip localization. However, it is worth noting that this variance may lack clinical significance, thereby supporting the value of localization use for identifying the clipped node. Our findings differ from those in the OMA study, which showed fewer nodes removed with TAD compared with SLN surgery without clip localization and removal (mean of 3 versus 4 nodes, respectively).^25^ However, the OMA study only included patients who converted to pN0 status, while ours included a high proportion of patients who remained pN+. Initial analyses from the ongoing TAXIS study showed that tailored axillary surgery in those with residual nodal disease excised a median of 4 lymph nodes (with interquartile range 3–5), which appears somewhat in line with our findings.^26^

Finally, we found no differences in EFS for those with or without a clip, or for those without successful clip retrieval. However, we acknowledge that our cohort of patients with clips placed had a lower burden of disease, potentially confounding our results. Yet, our finding is still consistent with recent studies demonstrating overall low nodal recurrence rates for patients with cN+ disease,^27,28^ a trend that remains true regardless of nodal clipping.^18,19^

Overall, our findings show that surgeons are increasingly utilizing nodal clipping in patients with cN+ disease prior to NAC, and this practice is associated with potential benefit of deescalation of axillary surgery without demonstrated harm thus far. Nonetheless, placing a clip in the node at time of diagnosis is potentially an additional procedure with associated financial costs, as is the localization procedure which is performed after NAC. If clip placements are performed at the time of needle biopsy, it likely presents minimal risk and a small amount of extra time for patients and radiologists/surgeons. The use of ultrasound-visible clips can facilitate localization with intraoperative ultrasound, but other factors such as visibility and ease of identification must be considered.^29^ The impact of various clips on magnetic resonance imaging (MRI) interpretation is also a consideration, particularly in the I-SPY2 trial, which employs serial MRI to assess response while on NAC. Advances in localization techniques will be essential as the use of TAD and tailored axillary surgical approaches increases.

These data from our multicenter study across the United States suggest that clip placement in patients with cN+ disease is increasingly common, possibly both for the purposes of improving the sensitivity of SLN surgery after NAC, and also to facilitate definitive axillary management even in patients with pN+ disease, consistent with tailored axillary surgery. While our study benefits from the multicenter nature and rigorous data collection in the setting of a prospective trial, there are numerous limitations to these analyses that must be acknowledged. First, the ISPY-2 trial was not designed to test surgical interventions. Accordingly, surgical procedures performed are subject to selection bias, and some information, including the use of clip placement, was collected retrospectively. Although we identified a strong association between clip placement and avoidance of ALND, even when adjusted for surgical year and cN category, we cannot conclude causation. As we do not know the decision-making process underlying management decisions, we can only postulate based on our observations. Future work will include surveying surgeons regarding axillary management decisions, particularly the omission of ALND for patients with pN+ disease despite the lack of prospective, randomized data supporting this approach. Importantly, data on the use of radiotherapy was not available for this analysis, which is a critical consideration when evaluating recurrence outcomes. Lastly, we were also limited by our mean follow-up time of 3.5 years, although all patients had molecularly high-risk breast cancer, which has a higher risk of earlier recurrence.

## Conclusion

Analysis of 801 patients with cN+ disease from a prospective NAC trial showed that clip placement in a positive axillary lymph node prior to NAC is increasingly common and is independently associated with SLN-only and facilitates avoidance of ALND with no negative impact on EFS. Clip placement in the node at the time of diagnosing cN+ disease may help tailor the surgical approach to the axilla.
